# Yawning and scratching contagion in wild spider monkeys (*Ateles geoffroyi*)

**DOI:** 10.1038/s41598-023-35693-5

**Published:** 2023-05-24

**Authors:** Sara Valdivieso-Cortadella, Chiara Bernardi-Gómez, Filippo Aureli, Miquel Llorente, Federica Amici

**Affiliations:** 1 Universitat de Girona, Fundació UdG: Innovació i Formació, 17003 Girona, Spain; 2 Instituto de Neuroetologia, Universidad Veracruzana, 91190 Xalapa-Enríquez, Mexico; 3 Research Centre in Evolutionary Anthropology and Palaeoecology, Liverpool John Moores University, Liverpool, L1 2SF UK; 4 Departament de Psicologia, Universitat de Girona, 17004 Girona, Spain; 5 Institute of Biology, Human Biology and Primate Cognition Group, University of Leipzig, 04109 Leipzig, Germany; 6 Department of Comparative Cultural Psychology, Max-Planck Institute for Evolutionary Anthropology, 04103 Leipzig, Germany

**Keywords:** Evolution, Psychology, Zoology

## Abstract

Behavioural contagion is a widespread phenomenon in animal species, which is thought to promote coordination and group cohesion. Among non-human primates, however, there is no evidence of behavioural contagion in Platyrrhines (i.e. primates from South and Central America) yet. Here, we investigated whether behavioural contagion is also present in this taxon, by assessing yawning and scratching contagion in a wild group (N = 49) of Geoffroy’s spider monkeys (*Ateles geoffroyi*). We conducted focal samples to examine whether individuals observing the triggering event (i.e. a naturally occurring yawning or scratching event in the group) would be more likely to yawn or scratch in the following 3 min, as compared to individuals who did not observe the triggering event. We ran generalized linear mixed models using a Bayesian approach, and found that the probability of yawning and scratching was higher for individuals observing others yawning and scratching, respectively, as compared to individuals who did not observe such an event. Behavioural contagion did not vary depending on the observer’s sex, kinship or relationship quality with the individual performing the triggering event. These findings provide the first evidence for yawning and scratching contagion in a wild group of spider monkeys, and importantly contribute to the debate about the evolutionary origins of behavioural contagion in primates.

## Introduction

In group-living animals, the ability to coordinate behaviour with other group members may provide individuals with crucial fitness benefits, by for instance promoting social cohesion and increasing the effectiveness of anti-predatory strategies^1–3^. Mechanisms that allow individuals to effectively coordinate their activities in the group include behavioural synchrony (when individuals react to an external stimulus in the same way) and behavioural contagion (when the perception of others’ behaviour automatically triggers a similar behaviour in the observers^4,5^). Although some authors consider behavioural contagion to be linked to emotional contagion, empathy and perhaps even theory of mind^6–9^, behavioural contagion can also be explained more parsimoniously. For example, individuals can unconsciously mimic others’ behaviour (chameleon effect)—a phenomenon that is also common in humans^10^.

Yawning is one of the most studied examples of behavioural contagion. Spontaneous yawning is widespread across vertebrates^11^, and is thought to serve different functions, from increasing blood and brain oxygen intake, to regulating brain temperature and maintaining attentional levels and shared alertness^11–14^, although none of these hypotheses have been yet fully confirmed. In humans, yawning can be easily triggered by seeing, hearing, reading or thinking about others’ yawning (i.e. contagious yawning^6,15,16^). Contagious yawning has also been shown in other species. For example, contagious yawning has been observed in captive budgerigars (*Melopsittacus undulates*^17^), wild elephant seals (*Mirounga leonina*^18^), domestic pigs (*Sus scrofa*^19^), captive wolves (*Canis lupus lupus*^20^), and domesticated dogs (*C. lupus familiaris*^7,21^). In non-human primates, contagious yawning has been shown in several Catharrine species, including captive chimpanzees (*Pan troglodytes*^9,22–25^), captive bonobos (*P. paniscus*^26,27^), captive orangutans (*Pongo pygmaeus*^28^), captive and wild geladas (*Theropithecus gelada*^8,29^), and captive stump-tailed macaques (*Macaca arctoides*^30^). Chimpanzees, for example, are more likely to yawn after observing videos of conspecifics yawning rather than not yawning^9,22^, even when 3D-animated yawning events are used^23^. However, not all tested species show contagious yawning. In primates, there has been no evidence for contagious yawning in Strepsirrhines (captive ring-tailed lemurs, *Lemur catta* and captive black-and-white ruffed lemurs, *Varecia variegata*^31^) until recently (wild indris, *Indri indri*^32^), and there is no evidence in Platyrrhines yet (captive common marmosets, *Callithrix jacchus*^33^).

In addition to yawning, other behaviours can spread across group members as a result of behavioural contagion. Scratching, for instance, is a self-directed behaviour that is considered a reliable measure of anxiety in primates^34–36^. Unlike yawning, very few studies have assessed the contagious effect of scratching^37^. In humans, scratching is triggered by listening to the word “itching”^38^ or itch-related sounds^39^, and by observing others scratching^40–42^. In non-human primates, contagious scratching has been shown in captive orangutans^37^, captive Japanese macaques (*Macaca fuscata*^43^), wild Tibetan macaques (*M. thibetana*^44^) and captive rhesus macaques (*M. mulatta*^45^). As in the case of yawning, however, there is no evidence for scratching contagion in Platyrrhines yet (captive common marmosets^33^).

The occurrence of behavioural contagion may vary across species, but also within species, as it is not necessarily a ubiquitous phenomenon across groups and individuals (e.g. Refs.^25,46^). Behavioural contagion can vary across group members depending on the relationship they have with the individual performing the triggering event, and/or their own individual characteristics. Studies on primates and other species found that yawning contagion is more likely between kin and individuals that have higher-quality relationships, as compared to non-kin and individuals having lower-quality relationships (captive primates: chimpanzees^9^, bonobos^26,27^ and geladas^8^; captive wolves^20^, dogs^47^ and domestic pigs^19^). However, other studies found no effect of kin and quality relationship on the probability of showing behavioural contagion (captive chimpanzees^24^, captive bonobos^46,48^, wild geladas^29^, dogs^21^). Moreover, some studies suggest that females observing a triggering event show shorter latencies to contagion (captive wolves^20^), or a higher likelihood of showing the same behaviour than males (humans^49,50^, captive bonobos^46^; but see wild geladas^29^). However, other studies found no sex bias in behavioural contagion (captive chimpanzees^9,23^, wild lemurs^32^, dogs^7^).

In this study, we aimed to investigate behavioural contagion in a wild group of Geoffroy’s spider monkeys (*Ateles geoffroyi*), a species with no pronounced sexual dimorphism in body and canine size^51^. In particular, we aimed to assess whether contagious yawning and scratching are present in Platyrrhines, and how their natural occurrence varies across individuals. First, we predicted that individuals observing a yawning or scratching event would be more likely to yawn or scratch, respectively, as compared to individuals who did not observe such events (Prediction 1). Second, we predicted that behavioural contagion would be more likely (a) between individuals with a higher-quality relationship, (b) between maternal kin, and (c) in female observers, as compared to individuals having a lower-quality relationship, non-kin and male observers (Prediction 2).

## Methods

### Study site and subjects

The study was carried out in the natural protected area Otoch Ma'ax Yetel Kooh, in the Yucatan Peninsula, Mexico, which consists of an old-growth, semi-evergreen medium forest with up to 25 m tree, successional forest, patches of younger regenerating forest and lakes^52^. We studied a group of Geoffroy’s spider monkeys living in the protected area, which were completely habituated to humans and could be individually recognized through their facial and body traits^53^. At the onset of the study (July 2021), the group consisted of 47 individuals: 7 adult males, 14 adult females, 5 subadult males, 1 subadult female, 2 juvenile males, 7 juvenile females, 6 infant males and 5 infant females (see Ref.^54^ for clarifications on age classes; Supplementary Table S1). Group size and composition changed during the study period, with 2 immigrant subadult females joining the group in August and September 2021 and 2 infant males being born right before the end of the study period (November 2021). Maternal kinship (i.e. mother–offspring dyads and maternal siblings) was known for all study subjects, thanks to the 25-year demographic records of the study group^53^.

### Ethics statement

The study was purely observational, and it implied no manipulation of the study subjects, who were already habituated to human observers since many years, at the onset of the study. Permit to conduct research was provided by the CONANP (Comision Nacional de Areas Naturales Protegidas) and SEMARNAT (Secretaría de Medio Ambiente y Recursos Naturales), and received the consent of the Mayan community that lives in the protected area. Our methods were in line with the American Society of Primatologists Ethical Principles for the Treatment of Nonhuman Primates, and followed the Code of Best Practices for Field Primatology published by the American Society of Primatologists. All methods are reported in accordance with ARRIVE guidelines (https://arriveguidelines.org).

### Data collection

We collected data from July to December 2021, 5 days a week, from 6 am to 13.30 pm, using 15‐min focal animal samples with continuous sampling^55^ for a total of 805 focal samples (mean ± SE: 4.12 ± 0.29 h per subject). Focal subjects were all the group members, except the 2 infants that were born at the end of the study period, although they could be recorded as partners during other group members’ focal observations (N = 49). Focal subjects were selected on a pseudorandomized basis (i.e. preparing a list with all the individuals in a randomized order, and starting focal observations from the first individual on the list, giving priority to focal subjects with fewer focal samples). No animal was sampled more than once a day, and individuals from the same family unit (i.e. mother–offspring dyads and maternal siblings) were sampled after at least 30 min from each other, to increase the independency of observations. Focal samples were recorded using CyberTracker on mobile devices (Blackview BV9700 PRO, Runbo F1 4G 5.5), with one observer (CBG or SVC) dictating the data and the other writing them into the device. Data collection started only after the two observers reached 80% inter-observer reliability for the coded behaviours (see below).

In each focal animal sample, we collected all occurrences of yawning (i.e. the individual makes a deep inspiration, followed by a lengthy, forceful expiration with simultaneous contraction of many skeletal muscle groups^12,15,56^) and scratching (i.e. the individual repetitively draws its nails on the skin with the fingertips^36,57,58^) performed by the focal animal. Every single yawning was recorded as a separate event. For scratching, a new event was recorded whenever scratching occurred after a break of at least 3 s from the previous scratching (to avoid multiple coding of scratching events belonging to the same scratching bout). Recorded yawning and scratching events were not associated with the production of signals in the vocal modality, so that behavioural contagion could only occur in the visual modality. Moreover, they were collected in the absence of evident disturbing events (e.g. presence of tourists in the area, recent aggression in the subgroup). Whenever the focal animal engaged in a yawning or scratching event (hereafter, triggering event), we recorded: (a) its time of occurrence; (b) the identity of all the individuals within 5 m from the focal animal (hereafter, partners); (c) whether the partners could see the focal animal producing the triggering event, as assessed based on the partners’ facial orientation (i.e. partners were considered to see the triggering event if they were directly facing the focal animal’s face, or if their face was turned up to a 45-degree angle); (d) whether partners yawned/scratched within 3 min from the corresponding triggering event (see Refs.^22,30,37^ for the choice of the time-frame), and if so, the latency between the triggering event and the yawning/scratching event; and (e) the distance between partners and focal animal when the triggering event took place (i.e. body contact, < 1 m and 1–5 m). Simultaneously recording of the behaviour of all the individuals within 5 m from the focal animal was possible thanks to the presence of three observers, and it allowed us to compare the behaviour of individuals experiencing very similar conditions (i.e. in the same area, at the same time), but crucially differing in whether they observed the triggering event. Triggering events were only those produced by the focal animal (e.g. if more than one partner yawned after the triggering event, they were still coded in relation to the triggering event and the focal animal). This implies that some partners might have observed group members other than the focal animal producing the same behaviour as the triggering event, thus being more likely to also perform it through behavioural contagion, despite being classified as not having observed the triggering event. Therefore, our inability to control for this potential effect might have biased against finding evidence for our Prediction 1, but could not lead to false positives.

To assess the quality of the relationships between group members, we collected all occurrences of grooming (i.e. manipulation of another individual’s fur with hands or mouth) and co-feeding (i.e. feeding on the same fruit species within 1 m from each other) that involved the focal animal, specifying the identity of the partner and the exact duration of the social interaction. Every 2 min, we also recorded the identity of all the individuals within 5 m from the focal animal (i.e. proximity). Outside the feeding context, spider monkeys were often widely distributed in the environment (personal observation), and we considered that 5-m proximity would better capture patterns of proximity between individuals.

### Data analyses

For each possible dyad of group members, we calculated the proportion of time (out of the observational effort for the two individuals, i.e. the total time in which each of the two individuals was visible during focal animal samples) that the individuals in the dyad spent grooming, the proportion of time they spent co-feeding, and the proportion of scan observations in which they were in proximity. We then rescaled each of these three measures to vary between 0 and 1 (i.e. assigning 0 to the lowest value of each measure, and 1 to the highest value, and proportionally rescaling all the intermediate values), and we calculated the mean of these rescaled values as a proxy of dyadic relationship quality (so that all measures varied between 0 and 1 and equally contributed to our proxy). The results obtained were confirmed when using 1 m-proximity (instead of 5 m-proximity).

We ran generalized linear mixed models^59^ in R^60^, with the brms package (version 2.16.3; Ref.^61^) that uses a Bayesian approach. We ran two sets of models: one for yawning and one for scratching. In the first set, we entered one line for each yawning event and partner within 5 m from the focal subject yawning (N = 285). As response variable, we entered whether the partner also yawned within the next 3 min (0/1), using a Bernoulli distribution with logit link. In the most complex model, we tested whether partners that observed the yawning event were more likely to yawn than those that did not observe the event, and whether this effect was modulated by social factors and individual characteristics (i.e. by the quality of their relationship, by maternal kinship and by the partner’s sex). We therefore included as predictor variables the three 2-way interactions of kinship (0/1), relationship quality and partner’s sex with the variable about whether the partner looked in the subject’s direction when the subject yawned (0/1). The inclusion of these interactions allowed us to specifically assess whether social factors and individual characteristics affect the probability of yawning when partners perceived the yawning event, as compared to when they did not. In this model, we also entered time of the day (i.e. the hour at which the triggering yawning occurred, as yawning production may follow a circadian pattern^62,63^) and distance between subject and partner (as triggering events may be more effective at smaller distances) as control variables. We considered 3 distance measures: body contact, < 1 m and 1–5 m. In addition, subject and partner identity were entered as random factors. The second less complex model was identical to the most complex one, except that we entered whether the partner observed the yawing event (as test predictor) and relationship quality, kinship and observer’s sex (as control variables) as main effects, and not in interaction. The least complex model only included the two control variables (time of the day and distance between subject and partner) and the two random factors, but not the main effects described above. The second set of models was identical to the first set, but the data-set included one line for each scratching event and partner within the 5 m from the individual scratching (N = 3803). As response variable, we entered whether the partner scratched within the next 3 min (0/1), using a Bernoulli distribution with logit link.

Within each set, we then compared all models with the approximate leave-one-out (loo) cross-validation in the loo package^64^, selecting the best model based on the difference (and standard error) between the expected log pointwise predictive densities (elpd) of the three models, which is a measure of the predictive ability of the models for new data^65^. For this purpose, we used the loo function in brms, which orders models based on their elpd, with a higher ratio between elpd difference and standard error indicating a stronger difference in fit between models. All models were run using flat priors, as we did not have any prior knowledge about the existence and strength of behavioural contagion in spider monkeys, which have never been studied before. We ran models with 4 chains in parallel (to increase the number of independent samples from our models and improve inference accuracy) and 2000 iterations each, half of them being warm-up samples that improve sampling efficiency. Warm-up samples are excluded from the target posterior distribution and adapt real samples to be immediately from the target distribution^66^. We conducted posterior predictive checks using the bayesplot package^67^. Convergence was suggested by Rhat estimates of 1.00 and a high effective number of samples in our models^66^. Pareto k estimates were very good (i.e. k < 0.5) for all models presented, and there were no collinearity issues (VIFs of best models: from 2.52 to 3.05). As some dyads were observed more than once, we also reran all models including the interaction of subject and partner identity as random factors. For the scratching data set, these models confirmed the results obtained without this more complex random factor structure. For the yawning data set, however, adding this more complex random factor structure led to convergence problems (i.e. Rhat > 1.00) and highly unreliable estimates (i.e. Pareto k estimates > 0.5, even when including moment matching corrections), likely due to the smaller sample size. Therefore, we decided to present the models with a simpler random structure.

## Results

### Yawning

**Figure 1:**
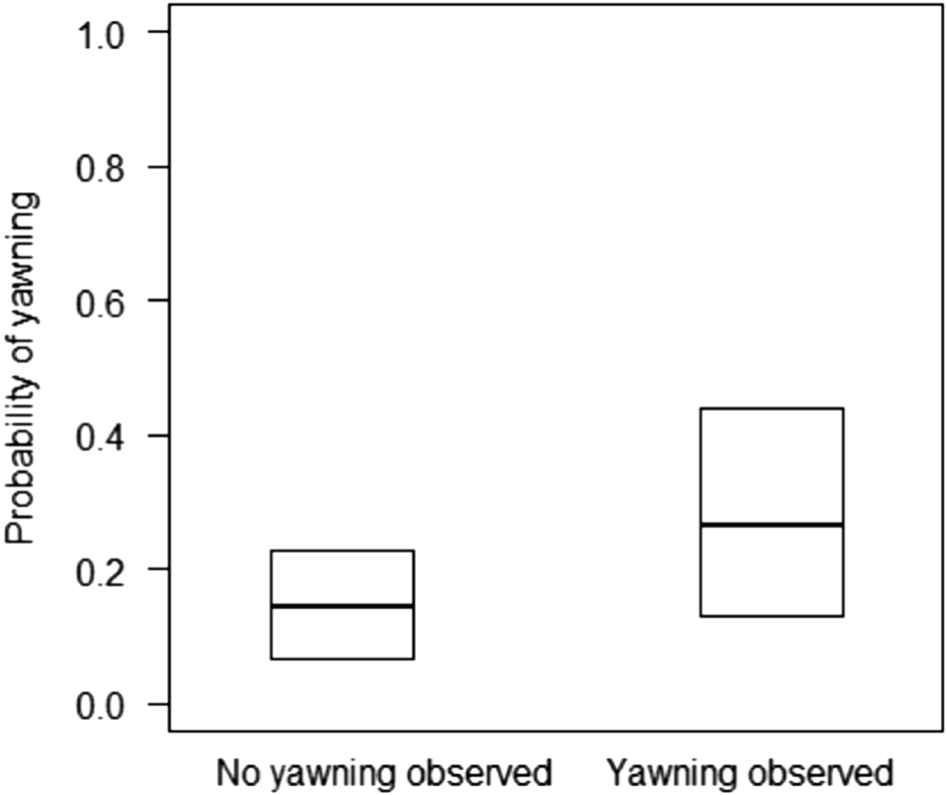
Thick lines represent the median estimated probability of individuals yawning after observing or not the yawning triggering event, back-transformed from the logit scale, and averaged over the level of maternal kinship and observer’s sex. Boxes represent the lower and upper 95% highest posterior density (HPD) interval probabilities.

We recorded yawning events by 35 focal animals. Partners yawned within 3 min after the triggering yawning event in 31.1% of the cases in which they observed the triggering yawning and 21.9% of the cases in which they did not (Fig. 1). The latency between the initial yawning event and the partner’s yawning was 62 ± 53 s (mean ± SD) for partners observing the triggering event. For the first set of models, the second less complex model had a slightly higher predictive ability for the yawning data than the most complex model (elpd difference: − 2.3 ± 2.6) and the least complex model (elpd difference: − 2.8 ± 3.0). Yawning by partners was more likely when they saw the focal subject yawning, as compared to when they did not (Fig. 1). In addition, females were more likely to yawn than males (Table 1). We could not run models including the interaction of subject and partner IDs as random factors, in addition to subject and partner IDs, because the models had convergence issues (i.e. Rhat > 1.00), likely due to the relatively small sample size.

**Table 1:** Estimate, standard deviation (SD) and two-sided 95% credible intervals (CIs) for each predictor of the best model in the first set (i.e. for yawning contagion).

Predictors	Estimate	SD	2.5 to 97.5% CI
Intercept	0.36	1.08	− 1.71 to 2.47
Observation of triggering yawning (0/1)	0.78	0.40	0.00 to 1.56
Relationship quality	− 1.78	1.91	− 5.77 to 1.64
Maternal kinship	− 0.09	0.74	− 1.49 to 1.42
Observer’s sex (male)	− 1.26	0.49	− 2.28 to − 0.37
Distance between partner & focal animal	− 0.14	0.09	− 0.31 to 0.04
Time of the day	− 0.10	0.13	− 0.36 to 0.16

CI intervals that include only positive or only negative values indicate that the predictor has a positive or negative effect on the response.

### Scratching

**Figure 2:**
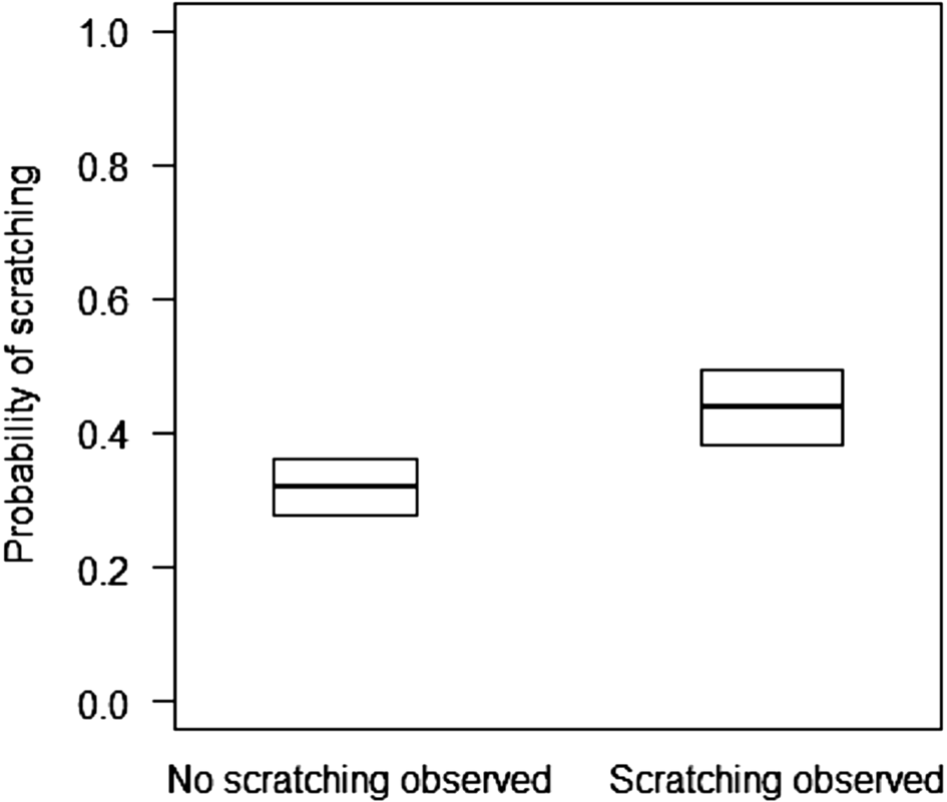
Thick lines represent the median estimated probability of individuals scratching after observing or not the scratching triggering event, back-transformed from the logit scale, and averaged over the level of maternal kinship and observer’s sex. Boxes represent the lower and upper 95% highest posterior density (HPD) interval probabilities.

We recorded scratching events by all 49 study subjects. Partners scratched within 3 min after the triggering scratching event in 49.8% of the cases in which they observed the triggering scratching and 32.1% of the cases in which they did not (Fig. 2). The latency between the initial scratching event and the partner’ scratching was 32 ± 28 s (mean ± SD) for partners observing the triggering event. Also for the second set of models, the second less complex model had a higher predictive ability for the scratching data than the more complex model (elpd difference: − 1.7 ± 1.7) and the simplest model (elpd difference: − 16.7 ± 6.5). Scratching by partners was more likely when they saw the focal subject scratching, as compared to when they did not (Fig. 2) and when their distance from the subject was smaller (Table 2). These results were confirmed by models including the interaction of subject and partner IDs as random factors, in addition to subject and partner IDs.

**Table 2:** Estimate, standard deviation (SD) and two-sided 95% credible intervals (CIs) for each predictor of the best model in the second set (i.e. for scratching contagion).

Predictors	Estimate	SD	2.5 to 97.5% CI
Intercept	− 0.05	0.23	− 0.51 to 0.40
Observation of triggering scratching (0/1)	0.52	0.09	0.35 to 0.69
Relationship quality	0.44	0.37	− 0.29 to 1.16
Maternal kinship	0.01	0.14	− 0.27 to 0.29
Observer’s sex (male)	− 0.20	0.16	− 0.52 to 0.11
Distance between partner & focal animal	− 0.12	0.02	− 0.15 to − 0.08
Time of the day	− 0.03	0.02	− 0.07 to 0.01

CI intervals that include only positive or only negative values indicate that the predictor has a positive or negative effect on the response.

## Discussion

Our study provided the first evidence for behavioural contagion in spider monkeys. In particular, we showed that individuals observing a group member yawning or scratching were more likely to yawn or scratch than individuals who did not observe such an event, in line with our Prediction 1. Therefore, our results show that yawning and scratching contagion are also present in Platyrrhines, and importantly contribute to the debate about the evolutionary origins of primate behavioural contagion. To date, yawning contagion has been shown in a variety of Catharrine species^8,9,22–29^, and more recently, in lemurs^32^. By providing evidence of yawning and scratching contagion also in Platyrrhines, our study provides further support to the hypothesis that behavioural contagion emerged before the evolutionary split between these taxa^68,69^. However, it is of course impossible to rule out that behavioural contagion independently evolved multiple times across taxa, perhaps in response to the specific socio-ecological conditions experienced by different species, groups and individuals. Indeed, this might explain why behavioural contagion is not present in some Catharrine species (e.g. gorillas (*Gorilla gorilla gorilla*): with video-recorded stimuli^70^), or in some groups and individuals of species that otherwise show behavioural contagion (see e.g. within-species variation in humans: with video-recorded stimuli^6^, with video-recorded and photo stimuli^50^; bonobos: with conspecifics^46^, and macaques: with video-recorded stimuli^30^).

In spider monkeys, the contagion effect occurred on average after 62 s from the triggering yawning event and after 32 s from the triggering scratching event. These results are in line with previous studies in other species showing that behavioural contagion takes place within the first minute after the triggering yawning event (with conspecifics: Refs.^26,27,46^), or within the second minute (with conspecifics: Refs.^29,71^; with video-recorded stimuli: Ref.^8^), and within the first 90 s after the triggering scratching event (with conspecifics: Refs.^37,45^). Moreover, our results show contagion rates similar to those found in the literature. In our study, the percentage of cases in which partners yawned after observing the triggering event was 31% (in contrast to 22% for individuals not observing it), as compared to 15–40% of contagion after yawning events in captive bonobos^27,46^, 5–20% in captive geladas^8^ and 15–45% in wild geladas^29^, all tested with conspecifics. Similarly, the percentage of cases in which partners scratched after observing the triggering event was 50% (in contrast to 32% for individuals not observing it), as compared to 10–25% of contagion after scratching events in captive orangutans (with conspecifics: Ref.^37^). At first sight, the percentage of cases in which partners yawned and scratched after observing no triggering events (22% and 32%, respectively) might appear unusually high. However, yawning and scratching can be caused by specific social and environmental conditions (e.g. uncertainty, low oxygen^12–15,34–36^), so that, individuals in spatial proximity, such as the partners within 5 m of the focal animal, are likely to co-experience. Therefore, partners were more likely to yawn and scratch when the focal animal yawned and scratched, regardless of having observed the triggering event. By directly comparing behaviour of partners exposed to the same environmental conditions within the same time window and only differing in whether they observed the triggering event, we thus ensured that differences between the two types of partners likely depended on behavioural contagion. Moreover, as explained in the Methods, it is possible that we might have partially underestimated the effect of behavioural contagion, as some partners might have observed group members other than the focal animal producing the same behaviour as the triggering event, thus being more likely to also perform it through behavioural contagion, despite being classified as not having observed the triggering event. Crucially, this could not lead to false positives, but rather biased against finding evidence for our Prediction 1.

Spider monkeys are characterized by a high degree of fission–fusion dynamics, with frequent changes in subgroup size and composition: therefore, group members may not be together for relatively long periods of time and social relationships might change during these periods^72^. Possibly, behavioural contagion might thus serve as a cognitively undemanding way for individuals to rapidly tune in to other group members upon fusions (see Refs.^73,74^), by mimicking others’ behaviour, synchronizing activities and ultimately promoting group coordination and social cohesion in the face of potentially important changes. Moreover, given that behavioural contagion is considered a precursor of other important social and cognitive skills, like emotional contagion, empathy or theory of mind^6–9,75^, it will be interesting to assess whether these skills are also present in this species.

In contrast to our Prediction 2, we did not find an effect of partner’s sex, maternal kinship and relationship quality on yawning and scratching contagion. We tested for this effect by including the interaction between these factors and whether the partner observed the triggering event in our most complex models, which however did not have a higher predictive ability than the other models. Females were not more likely to show yawning contagion than males in line with previous results in wild geladas (with conspecifics: Ref.^29^). Sex had however an effect on the occurrence of yawning (regardless of its spontaneous or contagious nature) with females being more likely to yawn than males. This may be related to the low sexual dimorphism in canine size in spider monkeys^51^ as males yawn more than females in sexually dimorphic non-human primates^76^ (e.g. Japanese macaques, long-tailed macaques (*M. fascicularis*^77^), stump-tailed macaques^78^, Sulawesi crested black macaques (*M. nigra*^79^), chacma baboons (*Papio ursinus*^80^)). Moreover, we found no evidence that behavioural contagion was higher between kin and individuals with a better-quality relationship, in contrast with what was found in other studies (with conspecifics: gelada baboons^8^, bonobos^26,27^; video-recorded stimuli: chimpanzees^9^). However, there are also studies that found no link between kinship and/or relationship quality and behavioural contagion. For example, chimpanzees did not yawn more frequently after watching yawning videos of familiar rather than unfamiliar conspecifics^22^, and in wild geladas yawning contagion was higher between individuals from different core units, who are less likely to engage in positive social interactions^29^. Similarly, Barbary macaques watching scratching videos paid more attention to familiar individuals with weaker rather than stronger social relationships^81^. The absence of evidence for an effect of relationship quality and kinship in our study indicates that in spider monkeys behavioural contagion might be better explained by emotional synchrony rather than emotional contagion, which is expected to play a role between socially close individuals^4,5^. However, the link between behavioural contagion and kinship/relationship quality is still unclear.

Overall, by providing the first evidence for yawning and scratching contagion in wild spider monkeys, and considering the recently findings in wild lemurs^32^, our study supports the hypothesis that behavioural contagion emerged before the split between Strepsirrhines and Haplorhines, and is therefore likely to be present across primate species. Moreover, our study opens up to new lines of investigation that might provide novel perspectives on the link between behavioural contagion and social complexity, such as whether individuals might more heavily rely on behavioural contagion to effectively tune in with other group members in species characterized by high levels of fission–fusion dynamics. Finally, our results confirm the use of wild settings as a powerful approach to study animal behaviour and cognition, since they provide large sample sizes and high ecological validity complementing controlled studies in captive settings.

## Supplementary Information

Supplementary Information.

## Data Availability

Data will be made available upon reasonable request to the corresponding author.
