# Supplementary material for: Yawning and scratching contagion in wild spider monkeys (Ateles geoffroyi)
Source: Sci Rep. 2023 May 24;13:8367. doi: 10.1038/s41598-023-35693-5 (PMC10209189; doi:10.1038/s41598-023-35693-5)
Supplement: Supplementary file 1 — Supplementary Information. [file 41598_2023_35693_MOESM1_ESM.docx]

**SUPPLEMENTARY MATERIAL**

**Table S1.** Detailed information about the study subjects, including their birth date, mother identity (when known), sex, age class, immigration date (when applicable), total number of focal samples conducted and hours of observation, from July 2021 through December 2021.


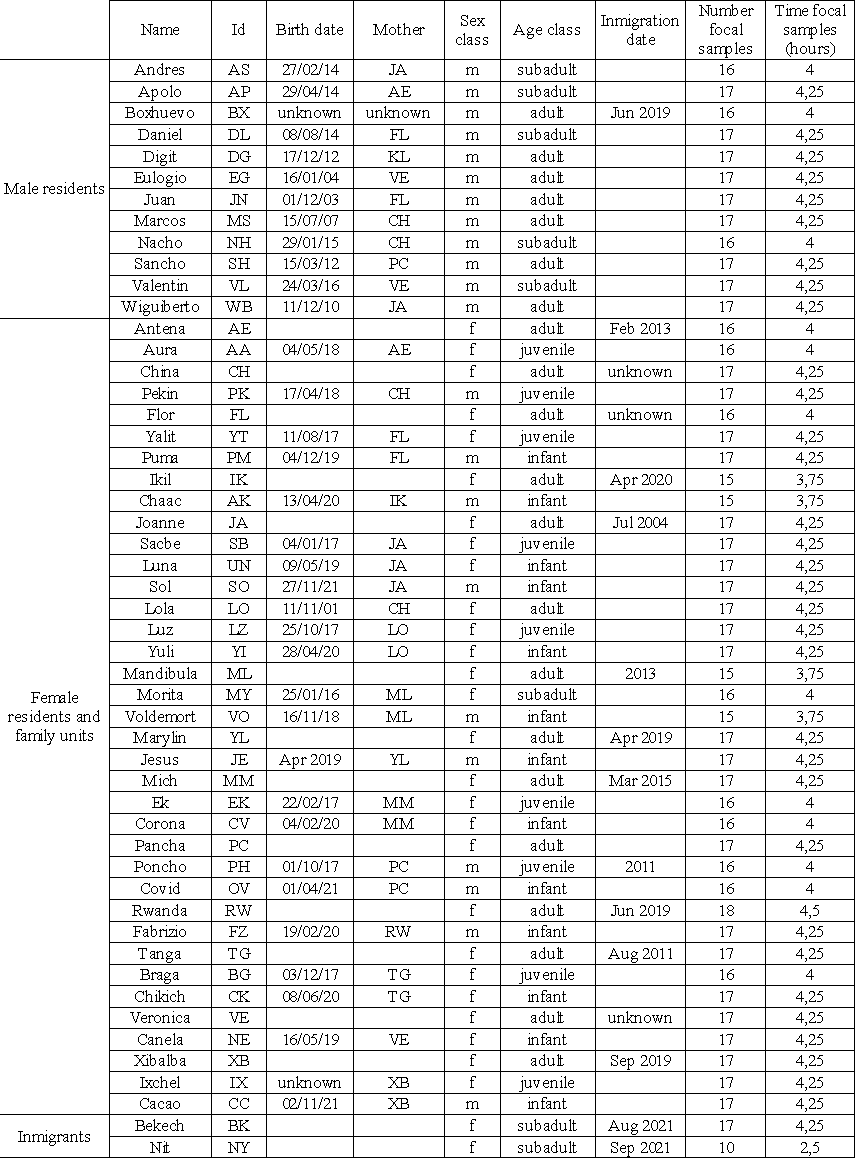


**Script used for the analyses**

library(brms)

library(tidyverse)

library(emmeans)

library(ggplot2)

library(DHARMa)

library(performance)

setwd("C...")

###### MODEL 1: yawn

xdata=read.table(file="Yawn.txt",header=T, sep="\t")

str(xdata)

hist(xdata$yawnsback1.no0)

res1=brm(yawnsback1.no0 ~ look1.no0 * (soc.ind + kin + f.sex) +

distance + hour + (1|sub) + (1|partner), data=xdata, family="bernoulli", seed=15)

m1=brm(yawnsback1.no0 ~ look1.no0 + soc.ind + kin + f.sex +

distance + hour + (1|sub) + (1|partner), data=xdata, family="bernoulli", seed=15)

null1=brm(yawnsback1.no0 ~

distance + hour + (1|sub) + (1|partner), data=xdata, family="bernoulli", seed=15)

res1 <- add_criterion(res1, "loo")

m1 <- add_criterion(m1, "loo")

null1 <- add_criterion(null1, "loo")

loo(res1, m1, null1, moment_match = TRUE)

summary(m1)

#CHECKS

check_collinearity(m1) # after removing interactions

point_preds <- fitted(m1)[, 1]

point_errs <- residuals(m1)[, 1]

qplot(point_preds, point_errs)

bayesplot package

pp_check(m1)

pp_check(m1, type = "error_hist", ndraws = 11)

###### MODEL 2: scratch

xdata=read.table(file="Scratch.txt",header=T, sep="\t")

str(xdata)

hist(xdata$scratchesback1.no0)

res2=brm(scratchesback1.no0 ~ look1.no0 * (soc.ind + kin + f.sex) +

distance + hour + (1|sub) + (1|partner), data=xdata, family="bernoulli", seed=15)

m2=brm(scratchesback1.no0 ~ look1.no0 + soc.ind + kin + f.sex +

distance + hour + (1|sub) + (1|partner), data=xdata, family="bernoulli", seed=15)

null2=brm(scratchesback1.no0 ~

distance + hour + (1|sub) + (1|partner), data=xdata, family="bernoulli", seed=15)

res2 <- add_criterion(res2, "loo")

m2 <- add_criterion(m2, "loo")

null2 <- add_criterion(null2, "loo")

loo(res2, m2, null2, moment_match = TRUE)

summary(m2)

#CHECKS

check_collinearity(m2) # after removing interactions

point_preds <- fitted(m2)[, 1]

point_errs <- residuals(m2)[, 1]

qplot(point_preds, point_errs)

bayesplot package

pp_check(m2)

pp_check(m2, type = "error_hist", ndraws = 11)
